# The Protective Effect of Cyanidin-3-Glucoside on Myocardial Ischemia-Reperfusion Injury through Ferroptosis

**DOI:** 10.1155/2021/8880141

**Published:** 2021-02-06

**Authors:** Xin Shan, Zhi-Yang Lv, Meng-Jiao Yin, Jing Chen, Jie Wang, Qi-Nan Wu

**Affiliations:** ^1^Jiangsu Collaborative Innovation Center of Chinese Medicinal Resources Industrialization, Nanjing University of Chinese Medicine, Nanjing, 210023 Jiangsu, China; ^2^Hanlin College, Nanjing University of Chinese Medicine, Taizhou, 225300 Jiangsu, China

## Abstract

This study was conducted to estimate the protective effect of Cyanidin-3-glucoside (C3G) on myocardial ischemia-reperfusion (IR) injury and to explore its mechanism. The rats were subjected to left anterior descending ligation and perfusion surgery. In vitro experiments were performed on H9c2 cells using the oxygen-glucose deprivation/reoxygenation (OGD/R) model. The results showed the administration of C3G reduced the infarction area, mitigated pathological alterations, inhibited ST segment elevation, and attenuated oxidative stress and ferroptosis-related protein expression. C3G also suppressed the expressions of USP19, Beclin1, NCOA4, and LC3II/LC3I. In addition, treatment with C3G relieved oxidative stress, downregulated LC3II/LC3I, reduced autophagosome number, downregulated TfR1 expression, and upregulated the expressions of FTH1 and GPX4 in OGD/R-induced H9c2 cells. C3G could inhibit the protein levels of USP19 and LC3II. C3G promoted K11-linked ubiquitination of Beclin1. Further evidence that C3G reduced ferroptosis and ameliorated myocardial I/R injury was demonstrated with the ferroptosis promoter RSL3. Taken together, C3G could be a potential agent to protect myocardium from myocardial I/R injury.

## 1. Introduction

Cardiovascular disease is a major health problem in industrialised and developing countries, and the high morbidity and mortality have brought heavy healthy and economic burden on society. Cardiac ischemia causes heart tissue damage and myocardial cell necrosis. Pharmacological treatments and early reperfusion therapies can ameliorate the damage; however, these therapies can also cause irreversible myocardial ischemia/reperfusion (I/R) injury, such as deterioration of cardiac function and arrhythmia [1]. Therefore, there is an urgent need to explore effective treatments to reduce myocardial I/R injury.

Ferroptosis is a programmed cell death mediator dependent on iron and ROS [2]. Ferroptosis has been identified in numerous disorders, including cancer, neurodegenerative diseases, and cardiovascular diseases. As a programmed cell death, ferroptosis induces various lipid hydroperoxide-related diseases, including myocardial I/R lesions [3]. Except for necrosis and ferroptosis, autophagy has been suggested to be an important regulator of ischemic/reperfusion dysfunction. Autophagy is an adaptive response to multiple stimuli through the decomposition of harmful proteins and damaged organelles. In myocardial I/R injury, mild autophagy can maintain the intracellular environment and improve the cell survival, but excessive autophagy can cause increased levels of intracellular oxidative stress [4, 5]. The relationship between autophagy and ferroptosis in cardiac ischemia-reperfusion has been limited reported.

Ubiquitination modification governs a number of cellular functions and signaling. Deubiquitinating enzymes (DUBs) reverse ubiquitination by cleaving ubiquitin from the substrate. Ubiquitin specific peptidase (USP) family is one of the DUB subfamilies, among which USP19 is a 150 kDa tail-anchored ubiquitin-specific protease. USP19 participates in the regulation of diverse diseases, such as obesity [5], cancer [6], and muscle waiting [7], and proved to be highly related to autophagy. However, whether USP19 can control myocardial I/R injury remains unclear.

Cyanidin-3-glucoside (C3G) is a member of the anthocyanin family which belongs to a subgroup of flavonoids. It is widely distributed in purple or red vegetables and fruits. Recent studies have demonstrated that C3G has anti-inflammatory, antioxidant, hepatoprotective, and cardioprotective effects [8, 9]. Škėmienė et al. proposed that anthocyanins protected the heart from ischemia/reperfusion-induced apoptosis and necrosis in rats [10]. C3G relieved the doxorubicin-induced cardiotoxicity in mice [11]. C3G also prevented cardiac hypertrophy and diastolic heart dysfunction [12]. Nevertheless, the effect of C3G on myocardial I/R injury has not been fully understood. The present study was to evaluate the pharmacological effect of C3G on myocardial I/R-induced rats and investigate its potential mechanism.

## 2. Experimental Methods

### 2.1. Reagent

Diltiazem (DIL) was purchased from Shanghai Xinyi Wanxiang Pharmaceutical Co., Ltd. (Shanghai, China). Cyanidin-3-glucoside (C3G, #C832095, with the purity ≥98%) was obtained from Macklin (Shanghai, China). The anti-USP19 (ab167059), anti-Beclin1 (ab210498), anti-Transferrin receptor 1 (TfR1, ab1086), anti-NCOA4 (ab86707), and anti-Ubiquitin (linkage-specific K11) antibodies were provided by Abcam (Cambridge, UK). Anti-ferritin heavy chain (FTH1, #3998) and anti-LC3I/II (#12741) antibodies were supplied from Cell Signaling Technology (Boston, USA). Anti-GAPDH antibody, Goat Anti-Mouse IgG (H + L) HRP second antibody, and Goat Anti-Rabbit IgG (H + L) HRP second antibody were produced by Affinity (Cincinnati, USA).

### 2.2. Animals and Experimental Protocols

Adult male Sprague Dawley (SD) rats weighing 260-280 g were purchased from Qinglongshan Animal Farm (Nanjing, China). The rats were housed under a 12 h light/dark cycle at 23 ± 2°C with 40%-60% humidity, and they had free access to standard food and water. All animal experimental procedures were approved by the Nanjing University of Traditional Chinese Medicine (Ethical number: No. 201910A246).

After a week of adaptation, the rats were randomly assigned into five groups (*n* = 8): (1) sham group, rats receiving saline gavage and sham surgery were used as control group; (2) I/R model group, rats receiving saline gavage and left anterior descending (LAD) ligation surgery were used as the model group; (3) C3G-10 group, I/R model plus intraperitoneal injection of 10 mg/kg C3G; (4) C3G-20 group, I/R model plus intraperitoneal injection of 20 mg/kg C3G; and (5) DIL group, I/R model plus oral administration of 20 mg/kg diltiazem. C3G and DIL were dissolved in DMSO and then diluted with saline so that the DMSO concentration was less than 0.1% (*v*/*v*). This procedure was continued for 7 days. Rats were anesthetized with 50 mg/kg sodium pentobarbital, followed by LAD ligation to occlude the coronary artery for 30 min before ischemia-reperfusion model of the myocardium was established by releasing the sutures for 2 h [13]. Simultaneously, the rats in the sham group were subjected to an open-chest step but did not continue with the LAD ligation.

A standard limb lead II electrocardiogram (ECG) was monitored continuously during the procedure. Immediately after the rat was sacrificed, the heart tissue was removed for subsequent experiments.

### 2.3. TTC Staining

2,3,5-Triphenyl-2H-tetrazolium chloride (TTC) staining was used to measure the area of myocardial infarction. After the heart had been washed three times with precooled saline, the left ventricle was cut transversely into five sections with the same thickness and stained with TTC at 37°C for 15 min. After staining, normal areas of the myocardium were stained red, while infarcted areas were left unstained. Infarct rate (%) = area of infarcted area/total area × 100%.

### 2.4. H&E Staining

The heart tissues were fixed in 4% paraformaldehyde, and the samples were then embedded in paraffin, cut into 4 *μ*m thick sections and stained using the hematoxylin-eosin staining method according to the protocol. The histopathological changes in these samples were observed under microscope.

### 2.5. Detection of ROS

The level of ROS was examined by dihydroethidium (DHE) fluorescent probes (D1008, Yuheng, China) in tissues and 2,7-dichlorodihydrofluorescein diacetate (DCFH-DA) fluorescent probes (D1002, Yuheng, China) in cells. For tissues, the slices were incubated with DHE probe at 37°C for 1 h. Representative photographs were taken with a fluorescence microscope, and fluorescence intensities were used to indicate ROS levels. For cells, DCFH-DA fluorescent probe solution at a concentration of 10 *μ*M was added to the treated cells and incubated in a cell incubator at 37°C for 20 min. The fluorescence intensity was measured using a flow cytometer with excitation/emission wavelengths of 488/525 nm, and the average fluorescence intensity was quantified.

### 2.6. Measurement of Superoxide Dismutase (SOD), Malondialdehyde (MDA), and Fe^2+^

Superoxide Dismutase (SOD) Assay Kit (A001-3-2, Jiancheng, China), Malondialdehyde (MDA) Assay Kit (A003-1-2, Jiancheng, China), and Iron Assay Kit (ab83366, Abcam) commercial kits were used to determine the relative levels of SOD and the relative levels of MDA and Fe^2+^, respectively. All procedures were conducted in accordance with the instructions of the kits.

### 2.7. USP19 Lentivirus Construction and Infection

Lentivirus particles carrying USP19 (Lv-USP19) and its empty vector (Lv-CON) were constructed by Nanjing Biowarrior Co., Ltd. The lentiviral vectors were transfected at a multiplicity of infection (MOI) of 10 into H9c2 cells. 24 h later, the culture medium was replaced for 48 h. Then, the infected H9c2 cells were screened with puromycin and cultured for further investigation. Besides, the cells transfected by USP19 lentivirus for 72 h were used for western blot to confirm the transfection efficiency.

### 2.8. Cell Culture and Grouping

To estimate the effect of C3G on H9C2 cells, the oxygen-glucose deprivation/reoxygenation (OGD/R) in vitro model was constructed. The H9C2 cells were donated from Nanjing University of Traditional Chinese Medicine University and cultured in DMEM medium containing 10% fetal bovine serum (FBS) and double antibiotics (penicillin and streptomycin, 100 *μ*g/mL : 100 *μ*g/mL) at 37°C.

H9c2 cells were treated with OGD/R to simulate *in vivo* I/R conditions. The cells were divided into five groups: control group, OGD/R group, 25-C3G group, 50-C3G group, and 100-C3G group. H9c2 cells at 5 × 10^4^ cells/well were seeded into six-well plates and incubated normally for 24 h; then, the medium was replaced with low-sugar serum-free DMEM; 25, 50, and 100 *μ*M C3G were added to the corresponding C3G administration groups, and the six-well plates were transferred to an incubator under hypoxic conditions (1% O_2_, 5% CO_2_, and 94% N_2_) for incubation at 37°C for 2 h. The cells were incubated normally for another 24 h. Cells in the control group were always cultured under normal conditions.

In experiment 3, the cells were divided into five groups: control group, OGD/R group, OGD/R + C3G (100 *μ*M) group, OGD/R + RSL (8 *μ*M) group, and OGD/R + RSL (8 *μ*M) + C3G (100 *μ*M) group. 5 × 10^4^ cells/well H9c2 cells were seeded into six-well plates for 24 h. Each group was treated according to the appropriate method and OGD/R procedure as above description. Afterwards, all the cells were harvested for further investigation.

### 2.9. Detection of Cell Viability

H9c2 cells at 5 × 10^3^ cells/well were seeded into the 96-well plate for 24 h. The cells were treated with different concentration of C3G (0, 25, 50, 100 *μ*M) or not and stimulated with OGD/R process. After treatment, the supernatant was discarded and coincubated 5 mg/mL 3-(4,5-dimethylthiazol-2-yl)-2,5-diphenyltetrazoliumbromide (MTT) at 37°C. 4 h later, DMSO was added to dissolve the purple formazan crystals. The optical density was determined at 570 nm by an automated microplate spectrophotometer.

### 2.10. Transmission Electron Microscope (TEM)

The cell samples were immersed in the TEM-specific fixed solution provided by Wuhan Seville Biotechnology Co., Ltd. (Wuhan, China) and then dehydrated in steps of 50%, 70%, 80%, 90%, 95%, and 100% acetone. After dehydration, the samples were embedded in 618 epoxy resin. Ultrathin sections (approximately 75 nm) were then cut with a diamond knife and placed on a copper grid coated with carbon film. The samples were stained by uranium acetate for 15 min, washed, and treated with lead citrate containing sodium hydroxide for 10 min. Finally, a transmission electron microscope was employed to observe the section.

### 2.11. Western Blot Analysis

The heart tissues and H9c2 cells were collected and homogenized by RIPA. The concentrations of the samples were determined using BCA Protein Assay Kit (BCA1-1KT, Merck, Germany) according to the manufacturer's instructions. Equal amount of protein was separated by SDS-PAGE and transferred onto PVDF membrane. Thereafter, the membrane was blocked by 5% skim milk at room temperature for 1 h and incubated with the primary antibody at 4°C overnight. After washing, the sample was incubated with horseradish peroxidase-conjugated IgG antibody at room temperature for 1.5 h. The blots were detected using ECL chemiluminescent system (Affinity, Cincinnati, USA) and fluorescent imaging system (ChampChemi, Beijing, China).

### 2.12. Coimmunoprecipitation (Co-IP)

After transfection, drug treatment, and OGD/R procedure, the cells were lysed with lysis buffer. The lysates were incubated with anti-Beclin1 antibody or anti-IgG antibody. The samples were precipitated with 50% protein A/G agarose bead solution and slightly shaked overnight at 4°C. Afterwards, the bound proteins were incubated with anti-K11-Ub antibody for western blot analysis as described above.

### 2.13. Statistical Analysis

The results were presented as mean ± SEM. Statistical significance was evaluated using one-way ANOVA with Tukey multiple comparison test. A value of *p* < 0.05 was regarded as statistically significant.

## 3. Results

### 3.1. Effect of C3G on I/R-Induced Myocardial Ischemia-Reperfusion

**Figure 1:**
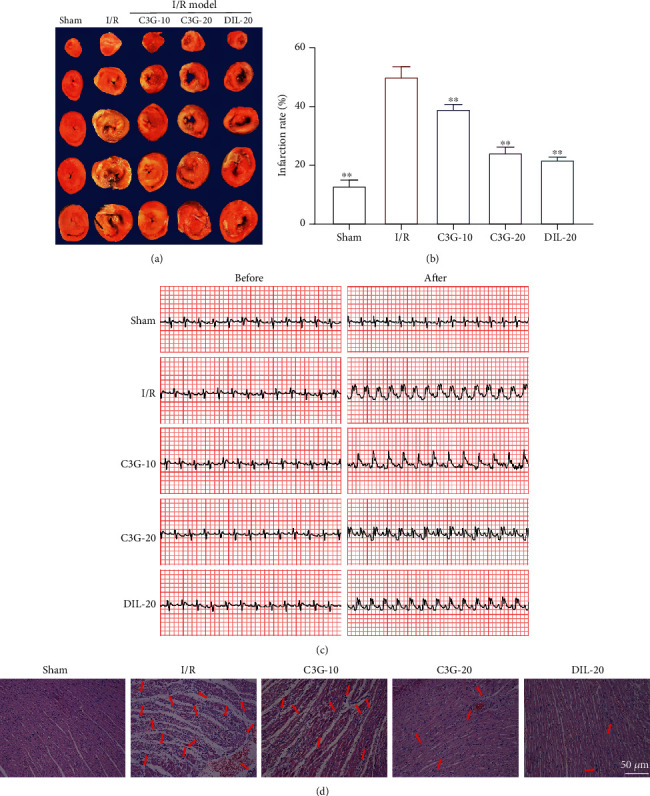
Effect of C3G on I/R-induced myocardial ischemia-reperfusion: (a) the TTC staining; (b) the ratio of infarction rate; (c) representative electrocardiograms before and after modeling; (d) the representative pictures of H&E staining. ^∗^*p* < 0.05, ^∗∗^*p* < 0.01 compared with the I/R group.

The infarct area of the ischemia heart was estimated using TTC staining. As shown in Figures 1(a) and 1(b), I/R stimulation indeed increased the infarct region compared with the sham group, while the administration with C3G notably reduced myocardial ischemia-reperfusion-induced infarction. Besides, the I/R stimulation led to the elevation of ST segment and variation of T wave. The C3G-20 and DIL groups conducted to the notable downregulation of ST segments, which were more efficient than that in the C3G-10 group (Figure 1(c)). The pathological alteration was evaluated through H&E staining. As presented in Figure 1(d), the clear myocardial structure, arranged fiber tissue, and scarce edema nor inflammatory cell infiltration were observed in the sham group. Whereas exposure to I/R contributed to the damaged myocardial structure, disarranged fibers, decreased myocardial cells, nuclear shrinkage, and inflammatory cell infiltration. The treatment with C3G and DIL evidently attenuated the histopathological changes as compared with the I/R group. The above results demonstrated that myocardial ischemia-reperfusion causes structural and functional injury to the heart and that C3G treatment could ameliorate the myocardial I/R injury.

### 3.2. Effect of C3G on Oxidative Stress Level and Fe^2+^ Content in Rats with Myocardial I/R

**Figure 2:**
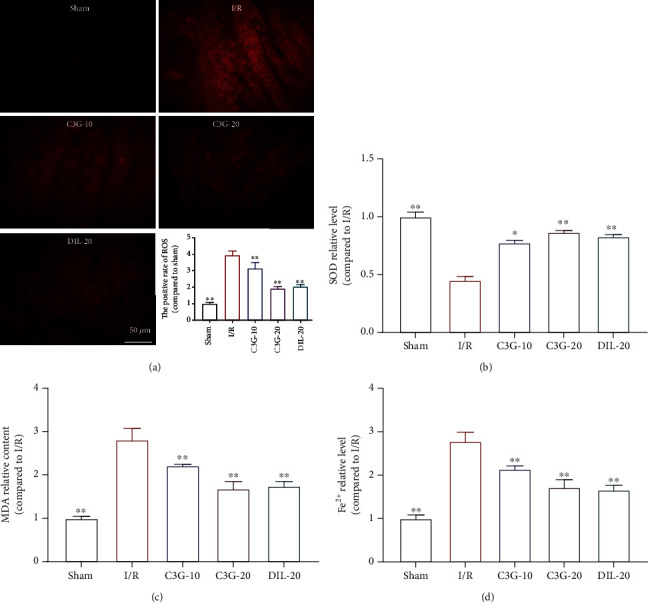
The effect of C3G on oxidative stress level and Fe^2+^ content in rats with myocardial I/R. (a) The level of ROS was analyzed by immunofluorescence and ratio of ROS positive cells/total cells. (b–d) The levels of SOD, MDA, and Fe^2+^ in heart tissues of different group. ^∗^*p* < 0.05, ^∗∗^*p* < 0.01 compared with the I/R group.

As shown in Figure 2(a), the elevated ROS level in the I/R group was significantly increased compared to the control group and then decreased by the treatment of C3G (*p* < 0.05). SOD and MDA decreased and increased in the I/R group, respectively, but returned towards normal levels in the C3G group (Figures 2(b) and 2(c)). These results suggest that myocardial I/R modeling increases the level of oxidative stress in the heart tissue, while C3G decreases this elevation. In addition, we also examined the contents of Fe^2+^ in the tissues and found that the trend in the changes in Fe^2+^ contents was consistent with the trend in oxidative stress levels (Figure 2(d)).

### 3.3. The Effect of C3G on USP19/Beclin1-Mediated Ferroptosis in Heart Tissues of I/R-Induced Myocardial Infarction

**Figure 3:**
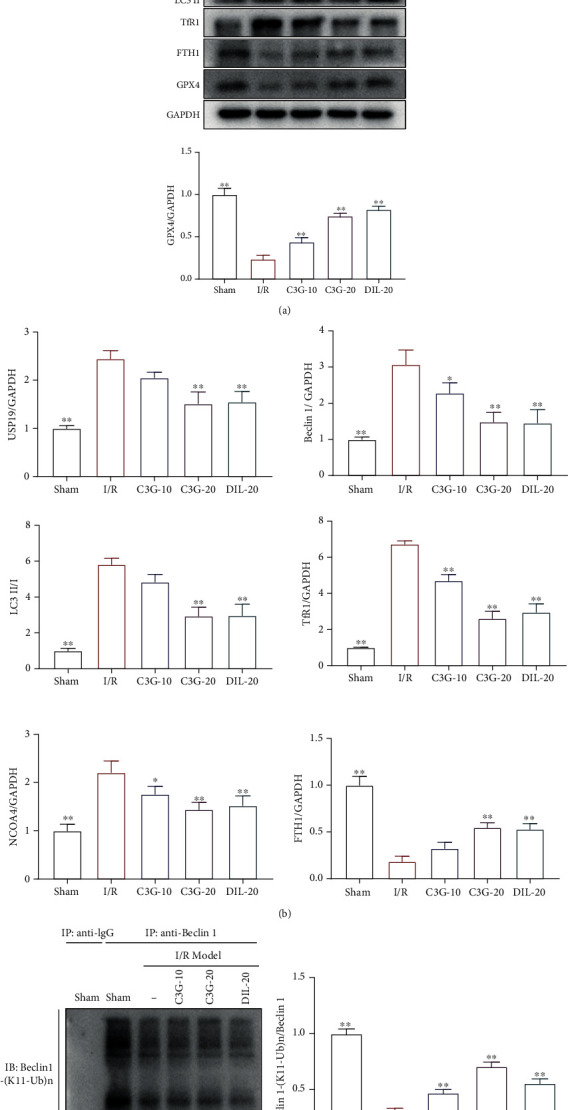
The effect of C3G on USP19/Beclin1-mediated ferroptosis in heart tissues of I/R-induced rats: (a) the effect of C3G on the expressions of USP19, Beclin1, NCOA4, LC3II/LC3I, GPX4, TfR1, and FTH1; (b) the relative levels of USP19, Beclin1, NCOA4, LC3II/LC3I, GPX4, TfR1, and FTH1 versus GAPDH; (c) the effect of C3G on the K11-linked deubiquitination by co-IP; (d) the relative levels of Beclin1-(K11-Ub)/Beclin1. ^∗^*p* < 0.05, ^∗∗^*p* < 0.01 compared with the I/R group.

To further investigate the potential mechanism of C3G on I/R-induced cardiac infarction, the expressions of USP19/Beclin1-mediated ferroptosis pathway-related proteins, including USP19, Beclin1, NCOA4, LC3II/LC3I, TfR1, and FTH1 were detected. As illustrated in Figures 3(a) and 3(b), the I/R procedure remarkably increased the protein levels of USP19, Beclin1, NCOA4, and LC3II/LC3I. On the contrary, protein levels of USP19 and Beclin1 were significantly decreased in the C3G-20 and DIL groups, with stronger therapeutic effect than those in the C3G-10 group. The C3G-20 and C3G-10 groups were effectively in reducing the augments of NCOA4 and LC3II/LC3I compared to those of I/R group. C3G treatment also clearly downregulated the protein expression of TfR1 and promoted the expression of FTH1 in I/R-induced cardiac tissues compared to the I/R group. Next, whether C3G influenced the K11-ubiqutination was detected by Co-IP. K11-ubiquitination was observed in the sham group; nonetheless, the I/R stimulation evidently blocked the combination between K11-ubiquitin and Beclin1, indicating that I/R-induced myocardial ischemia might be associated with deubiquitination. The administration of C3G could restore the ubiquitination of Beclin1 (Figures 3(c) and 3(d)). The data implied that the protective effect of C3G against I/R-induced myocardial ischemia may be relevant for USP19/Beclin1-dependent ferroptosis.

### 3.4. The Effect of C3G on Oxidative Stress Level and Fe^2+^ Content in H9c2 Cells

**Figure 4:**
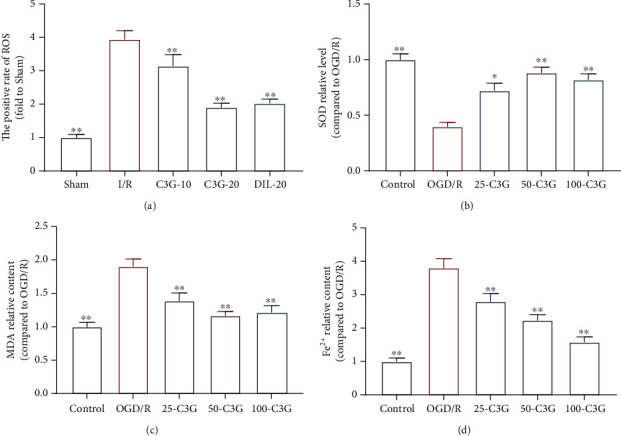
The effect of C3G on oxidative stress level and Fe^2+^ content in OGD/R-induce H9c2 cells: (a) the relative level of ROS; (b–d) the levels of SOD, MDA, and Fe^2+^ in cells of different group. ^∗^*p* < 0.05, ^∗∗^*p* < 0.01 compared with the I/R group.

OGD/R modeling caused a distinct increase in number of DCF^+^ cell, indicating an increase in ROS. Nevertheless, the treatment with C3G apparently reduced ROS level compared to that of the OGD/R group (Figure 4(a)). The exposure to OGD/R process dramatically promoted of the increase of MDA and Fe^2+^ and the reduction of SOD activity. In contrast, treatment with C3G (25 *μ*M, 50 *μ*M, 100 *μ*M) conspicuously increased SOD activity and decreased MDA content (Figures 4(b) and 4(c)). In addition, treatment with C3G reduced Fe^2+^ content in a concentration-dependent manner (Figure 4(d)). The results proved that C3G could reduce OGD/R-induced increases in oxidative stress levels and Fe^2+^ content in H9c2 cells.

### 3.5. The Effect of C3G on Autophagy of OGD/R-Induced H9c2 Cells

**Figure 5:**
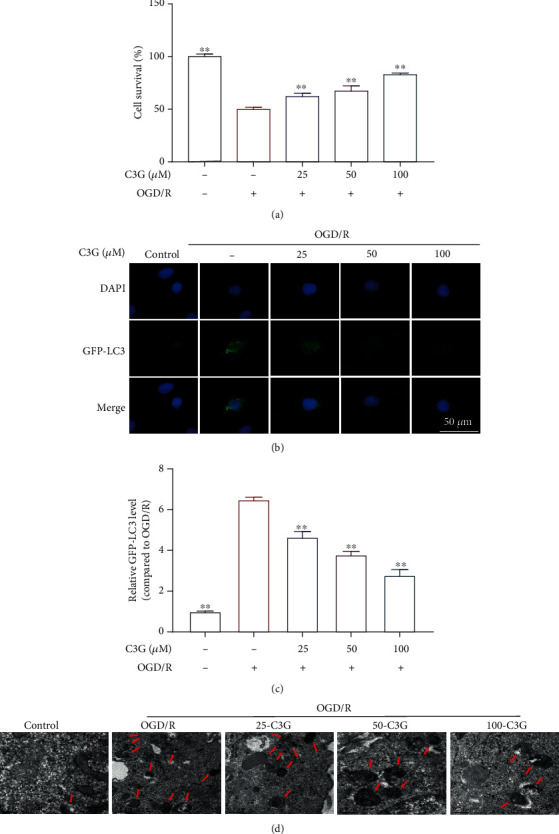
C3G increases cell viability and reduces autophagy. (a) The viability of cell in different group. (b) Representative images of the LC3 expression detected by immunofluorescence. (c) Relative fluorescence intensity of GFP-LC3. (d) The number of autophagosome was observed by transmission electron microscope. ^∗^*p* < 0.05, ^∗∗^*p* < 0.01 compared with the OGD/R group.

The MTT assay confirmed that C3G treatment could increase the viability of cell (Figure 5(a)). The immunofluorescence results showed that LC3B expression was upregulated in the OGD/R group, which was apparently downregulated in C3G-treated H9c2 cells (Figures 5(b) and 5(c)). Transmission electron microscope was employed to examine the autophagosome in H9c2 cells. As visualized in Figure 5(d), the number of autophagosomes was increased after OGD/R progression was observed compared with the control group. Whereas, incubation with C3G (25 *μ*M, 50 *μ*M, 100 *μ*M) led to the reduction of autophagosome number. Our results suggested that the protective effect of C3G on OGD/R-exposed H9c2 cells might be related to the inhibition of autophagy.

### 3.6. The Effect of C3G on USP19/Beclin1-Mediated Ferroptosis in OGD/R-Induced H9c2 Cells

In vitro, we explored the mechanism of C3G in OGD/R-induced H9c2 cells. As expected, the OGD/R model resulted in the upregulation of USP19, Beclin1, NCOA4, and LC3II/LC3I. The treatment with C3G (25 *μ*M, 50 *μ*M, 100 *μ*M) downregulated the expressions of USP19 and LC3II/LC3I in a concentration-dependent manner. The C3G treatment also reduced the protein levels of Beclin1 and NCOA4 ferroptosis.

**Figure 6:**
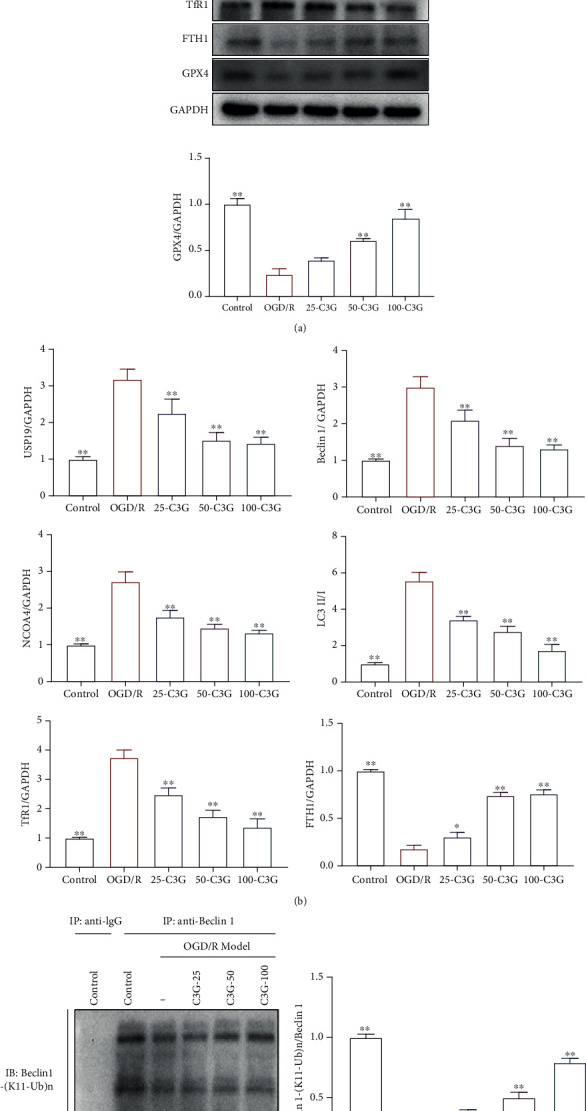
The effect of C3G on USP19/Beclin1-mediated ferroptosis in OGD/R-induced H9c2 cells: (a) the effect of C3G on the expressions of USP19, Beclin1, NCOA4, LC3II/LC3I, GPX4, TfR1, and FTH1; (b) the relative levels of USP19, Beclin1, NCOA4, LC3II/LC3I, GPX4, TfR1, and FTH1 versus GAPDH; (c) the effect of C3G on the K11-linked deubiquitination by co-IP; (d) the relative levels of Beclin1-(K11-Ub)/Beclin1. ^∗^*p* < 0.05, ^∗∗^*p* < 0.01 compared with the OGD/R group.

The effects of C3G on the expressions of TfR1 and FTH1 were measured. As observed in Figures 6(a) and 6(b), the TfR1 expression was upregulated, and the FTH1 expression was downregulated compared with the control group. The incubation with C3G (25 *μ*M, 50 *μ*M, 100 *μ*M) restored the changes in a concentration-dependent manner.

Furthermore, whether C3G altered the ubiquitination was also assessed *in vitro*. Ubiquitination of Beclin1 was reduced in the OGD/R group compared to the control group, indicating that Beclin1 levels were higher in OGD/R than in the control group. However, the C3G (25 *μ*M, 50 *μ*M, 100 *μ*M) incubation promoted the ubiquitination of Beclin1 (Figures 6(c) and 6(d)). Our data proposed that the effect of C3G on OGD/R-induced ferroptosis might be controlled by USP19/Beclin1-induced autophagy.

### 3.7. The Role of USP19 in the C3G-Mediated Protective Effect and Ferroptosis on OGD/R-Induced H9c2 Cells

**Figure 7:**
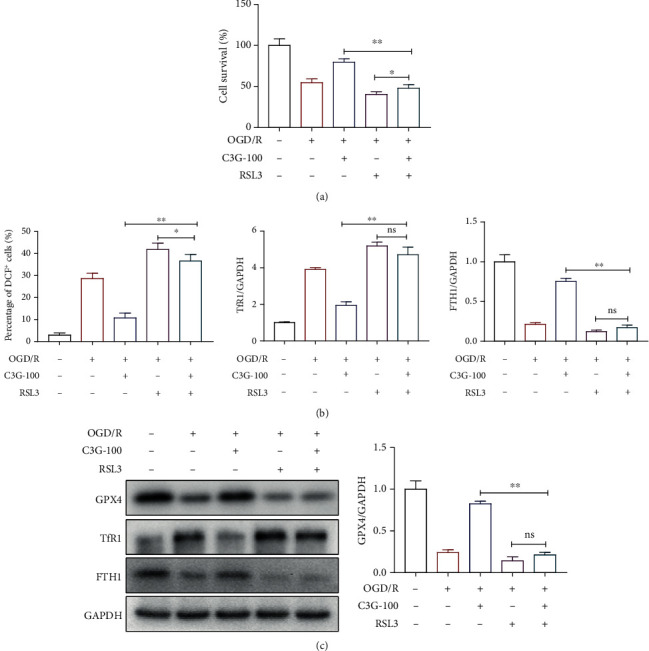
The role of C3G in RSL3-regulated autophagy. (a) C3G increased the viability of cells reduced by RSL3. (b) C3G reduced ROS levels that were increased by RSL3. (c) C3G inversely regulated the expression levels of iron death-related proteins that were altered by RSL3. When comparing the two sets of data, ^∗^*p* < 0.05 means significant, and ns means not significant.

In conjunction with the previous results, RSL3, a GPX4 inhibitor, was used to further investigate the protective role of C3G in myocardial ischemia-reperfusion.  Figure 7(a) shows that the cell viability was significantly increased in the C3G-100 group compared to the C3G-100 + RSL3 in the OGD/R modeling situation and was also significantly lower in the OGD/R + RSL3 group than in the OGD/R + C3G-100 + RSL3 group (*p* < 0.05). RSL3 effectively increased the level of ROS compared to the OGD/R + RSL3 group, and treatment with C3G significantly reduced the level of ROS (*p* < 0.05, Figure 7(b)). In Figure 7(c), OGD/R stimulation significantly increased the expression of TfR1 and decreased the expression of GPX4 and FTH1, a trend that was further promoted by the addition of RSL3, indicating that RSL3 promoted ferroptosis. In contrast, treatment with C3G decreased the TfR1 expression and promoted the GPX4 and FTH1 expression. Notably, the use of C3G effectively blocked the promoting effect of RSL3 on ferroptosis.

## 4. Discussion

The antioxidant effects of C3G can be used to treat diseases, and it was reported that C3G modulated oxidation dependent on iron [14]. C3G ameliorated ferroptosis via histone ubiquitination and oxidative mitigation [15]. These literatures indicated that C3G might exhibit its pharmacological effect through ferroptosis and ubiquitination modification. Our results demonstrated that C3G administration reduced cardiac infarction area, restrained pathological damage, and inhibited ST-segment elevation, which indicated that C3G treatment effectively prevented myocardial I/R lesion.

As a degradative process crucial in cellular physiology and pathology, autophagy is activated in the pathogenesis of myocardial I/R damage. Autophagy is a catabolic process implicated with the impaired proteins and organelles, characterised primarily by the generation of autophagosomes and degradation of lysosome, which is an essential pathway to maintain cell homeostasis. Under stressful conditions of energy deficiency or hypoxia, autophagy is initiated to clear the toxic events and facilitate cell survival [16]. Autophagy accelerates availability of iron in the hypoxic environment. It is noteworthy that slight autophagy improves cell survival, while excessive autophagy is detrimental during reperfusion and is attributed to the cellular constituent consumption [17]. The upregulation of autophagy during myocardial ischemia-reperfusion may be related to the stimulus conditions (duration of ischemia). Study has reported that in mice undergoing LAD surgery, autophagic flux increased, and damaged proteins were cleared within 0-6 h of reperfusion, and after 6 h, autophagic flux decreased, but the accumulation of autophagic vesicles was more pronounced, indicating that autophagy at this time was no longer able to play a role in clearing damaged proteins and was in a state of excessive autophagy to the cells [18]. The results of in vitro experiment showed that the expression of Beclin1 was upregulated in hypoxia/reoxygenation-induced H9c2 cells, implying an increase in autophagy, which was consistent with previous study [19]. Results from in vitro and in vivo experiments showed that C3G suppressed the expression levels of Beclin1 and LC3II in I/R-induced cardiac tissues and OGD/R-induced H9c2 cells and that inhibition of autophagy was benefit to the I/R model [20]. Therefore, autophagy was the key event in the ameliorated effect of C3G on myocardial I/R injury.

Beclin1 activity is modulated by a variety of posttranslational modifications including ubiquitination. USP is the largest family of DUBs controlling the reversible process of ubiquitination and is a target of the unfolded protein response (UPR) [21]. Mounting evidence indicated that USP19 drives numerous cellular process. An important role for USP19 in the innate immune response has now been reported [22], with USP19 blunting pathological cardiac hypertrophy by inhibiting the inflammatory response [23]. Increasing literature indicated the correlation between USP family and autophagy. The deletion of USP14 can activate endoplasmic reticulum stress-mediated autophagy [24]. USP13 knockdown increases p-tau ubiquitination via the 20S proteasome and p-tau clearance via autophagy [25]. USP8 directly deubiquitinates SQSTM1/p62 and blocks autophagy [26]. It is noteworthy that USP19 influences the ubiquitination of Beclin1, hence accelerating the production of autophagosome. USP19 stabilizes Beclin1 at residue K437 through a K11-linked polyubiquitin chains [27]. Previous investigators also confirmed that USP19 cleaves the K11-linked ubiquitin chains of Beclin1 in antiviral immunization and prevents its degradation via the proteasome. Our data indicated that the USP19 expression was upregulated in the I/R or OGD/R stimulation group, whereas C3G treatment downregulated the USP19 expression and blocked the K11-linked deubiquitination of Beclin1. The overexpression of USP19 by lentivirus showed that USP19 was closely associated with C3G-treated autophagy and ferroptosis.

It is acknowledged that lipid peroxidation and iron accumulation lead to ferroptosis [28]. Ferroptosis can cause cell death without the activation of caspases. Previous study has showed that expanded cardiomyopathy occurs in ferroportin-deficient cardiomyocytes [29]. As a key member of iron metabolism-associated genes, FTH1 is responsible for Fe^2+^ storage; TfR1 is responsible for controlling the Fe^3+^ transport across the cell membrane and maintaining intracellular iron homeostasis [30]. In our study, the level of ROS accumulation and iron content was increased in ex vivo models after I/R modeling. Iron overload is the key reason of myocardial cell injury [31]. Previous literature proved that the inhibition of ferroptosis exhibited a protective effect on myocardial I/R-induced rats and OGD/R H9c2 cells [32]. Our data demonstrated that C3G treatment was effective in alleviating oxidative stress, reducing Fe^2+^ content, downregulating the TfR1 expression, and upregulating the FTH1 expression both in cells and tissues, all confirming that C3G could reduce oxidative stress levels and decrease Fe^2+^ accumulation, both *in vivo* and *in vitro*.

## 5. Conclusion

In conclusion, our results displayed that C3G attenuated myocardial I/R injury by inhibiting ferroptosis both *in vivo* and *in vitro*. The C3G treatment prevented K11-linked deubiquitination of Beclin1. Further investigation with transgenic mice is warranted in the future.

## Data Availability

The data used to support the findings of this study are available from the corresponding author upon request.

## Abbreviations

C3G: Cyanidin-3-glucoside
Co-IP: Coimmunoprecipitation
DHE: Dihydroethidium
DIL: Diltiazem
DUBs: Deubiquitinating enzymes
ECG: Electrocardiogram
FBS: Fetal bovine serum
FTH1: Ferritin heavy chain
IR: Ischemia-reperfusion
MOI: Multiplicity of infection
MTT: 3-(4,5-Dimethylthiazol-2-yl)-2,5-diphenyltetrazoliumbromide
NCOA4: Nuclear receptor coactivator 4
OE: Overexpression
OGD/R: Oxygen-glucose deprivation/reoxygenation
ROS: Reactive oxygen species
SD: Sprague Dawley
TEM: Transmission electron microscope
TfR1: Transferrin receptor
TTC: 2,3,5-Triphenyl-2H-tetrazolium chloride
UPR: Unfolded protein response
USP19: Ubiquitin-specific protease 19.
